# Electromagnetic guided bedside or endoscopic placement of nasoenteral feeding tubes in surgical patients (CORE trial): study protocol for a randomized controlled trial

**DOI:** 10.1186/s13063-015-0633-1

**Published:** 2015-03-26

**Authors:** Arja Gerritsen, Thijs de Rooij, Marcel G Dijkgraaf, Olivier R Busch, Jacques J Bergman, Dirk T Ubbink, Peter van Duijvendijk, G Willemien Erkelens, I Quintus Molenaar, Jan F Monkelbaan, Camiel Rosman, Adriaan C Tan, Philip M Kruyt, Dirk Jan Bac, Elisabeth M Mathus-Vliegen, Marc G Besselink

**Affiliations:** Department of Surgery, Academic Medical Center, PO Box 22660, , 1100 DD Amsterdam, the Netherlands; Department of Surgery, University Medical Center Utrecht, PO Box 85500, , 3508 GA Utrecht, the Netherlands; Clinical Research Unit, Academic Medical Center, PO Box 22660, 1100 DD Amsterdam, the Netherlands; Department of Gastroenterology and Hepatology, Academic Medical Center, PO Box 22660, 1100 DD Amsterdam, the Netherlands; Department of Surgery, Gelre Hospital, PO Box 9014, 7300 DS Apeldoorn, the Netherlands; Department of Gastroenterology, Gelre Hospital, PO Box 9014, 7300 DS Apeldoorn, the Netherlands; Department of Gastroenterology and Hepatology, University Medical Center Utrecht, PO Box 85500, 3508 GA Utrecht, the Netherlands; Department of Surgery, Canisius Wilhelmina Hospital, PO Box 9015, 6500 GS Nijmegen, the Netherlands; Department of Gastroenterology, Canisius Wilhelmina Hospital, PO Box 9015, 6500 GS Nijmegen, the Netherlands; Department of Surgery, Hospital Gelderse Vallei, PO Box 9025, 6710 HN Ede, the Netherlands; Department of Gastroenterology, Hospital Gelderse Vallei, PO Box 9025, 6710 HN Ede, the Netherlands

**Keywords:** Nasoenteral feeding, Post-pyloric feeding, Feeding tubes, Enteral feeding, Enteral nutrition, Electromagnetic guidance, Endoscopy, Surgery, Gastroparesis

## Abstract

**Background:**

Gastroparesis is common in surgical patients and frequently leads to the need for enteral tube feeding. Nasoenteral feeding tubes are usually placed endoscopically by gastroenterologists, but this procedure is relatively cumbersome for patients and labor-intensive for hospital staff. Electromagnetic (EM) guided bedside placement of nasoenteral feeding tubes by nurses may reduce patient discomfort, workload and costs, but randomized studies are lacking, especially in surgical patients. We hypothesize that EM guided bedside placement of nasoenteral feeding tubes is at least as effective as endoscopic placement in surgical patients, at lower costs.

**Methods/Design:**

The CORE trial is an investigator-initiated, parallel-group, pragmatic, multicenter randomized controlled non-inferiority trial. A total of 154 patients admitted to gastrointestinal surgical wards in five hospitals, requiring nasoenteral feeding, will be randomly allocated to undergo EM guided or endoscopic nasoenteral feeding tube placement. Primary outcome is reinsertion of the feeding tube, defined as the insertion of an endoscope or tube in the nose/mouth and esophagus for (re)placement of the feeding tube (e.g. after failed initial placement or dislodgement or blockage of the tube). Secondary outcomes include patient-reported outcomes, costs and tube (placement) related complications.

**Discussion:**

The CORE trial is designed to generate evidence on the effectiveness of EM guided placement of nasoenteral feeding tubes in surgical patients and the impact on costs as compared to endoscopic placement. The trial potentially offers a strong argument for wider implementation of this technique as method of choice for placement of nasoenteral feeding tubes.

**Trial registration:**

Dutch Trial Register: NTR4420, date registered 5-feb-2014

**Electronic supplementary material:**

The online version of this article (doi:10.1186/s13063-015-0633-1) contains supplementary material, which is available to authorized users.

## Background

Gastroparesis, or delayed gastric emptying, occurs in approximately 2% of surgical patients [1,2]. It is caused by the disease itself, an operation, postoperative complications such as intra-abdominal infection, or concomitant underlying diseases such as diabetes. Patients suffering from gastroparesis do not tolerate a normal diet and may eventually become malnourished, which negatively affects clinical outcomes [3-5]. Therefore, in patients who are not able to achieve at least 50% of their daily required caloric intake for several consecutive days, it is common practice to place a nasoenteral feeding tube to deliver enteral nutrition. Parenteral nutrition is only used when enteral nutrition is contraindicated, not feasible or not sufficient in delivering nutrient needs, as parenteral nutrition has shown to be associated with an increase in infectious complications and higher costs [6].

Nasoenteral feeding tube placement can be challenging, especially in patients with gastroparesis. Blind placement is usually unsuccessful and may lead to complications such as pneumothorax and pneumonia due to inadvertent placement in the bronchus in over 2% of placement attempts [7].

Therefore, nasoenteral feeding tubes are usually placed endoscopically, regularly followed by abdominal radiography to confirm a correct position of the tube. Endoscopic feeding tube placement requires fasting and patient transportation between the clinical ward, endoscopy and radiology departments. The procedure is also relatively expensive as it has to be performed by a gastroenterologist and one or two endoscopy nurses.

Hence, both patients and hospital staff perceive endoscopic tube placement as uncomfortable and cumbersome. Furthermore, patients may need several repeated procedures during hospital admission. We noticed a 34% dislodgement rate of nasoenteral feeding tubes within the first week after placement [8]. Some 50% of these dislodged tubes require replacement, which not only leads to additional discomfort for the patient and temporary decrease in caloric intake, but also to an increased workload for nursing and medical staff, and additional costs.

Electromagnetic (EM) guided placement of nasoenteral feeding tubes is suggested to be less discomforting to the patient and seems cost-saving compared with endoscopy, as it can be performed on the clinical ward at the patient’s bedside by a specialized nurse. Confirmation of the tube’s position on an abdominal radiograph is unnecessary and repositioning of a tube that has dislodged into the stomach can be done with the stylet without the need for a fully repeated procedure [9-12]. Implementation of this technique requires training of nurses (e.g. a full-day training session, ideally followed by a number of supervised placement procedures) and purchasing the equipment ($ 7925) [10,13].

A recent systematic review by our group showed that EM guided tube placement in adult patients is not inferior to endoscopic placement regarding efficacy and safety in patients with normal upper gastrointestinal (GI) anatomy [14]. Success rates were similar and only minor complications such as epistaxis and dislodgement or blockage of the tube were reported. However, the available evidence is of moderate quality and lacks data on patient-reported outcomes and costs. Moreover, studies in surgical patients, especially those with an altered upper GI anatomy after surgery, are scarce. In our previous retrospective cohort study, EM guided placement was successful in 58% of patients with an altered upper GI anatomy, which seems low, but may be acceptable given the potential benefits for the patient [15]. A well-designed pragmatic, multicenter, randomized controlled trial is therefore needed to assess the true effectiveness and benefits of EM guided nasoenteral feeding tube placement in the surgical population.

The aim of the CORE trial is to determine the effectiveness of EM guided bedside placement as compared to endoscopic placement of a nasoenteral feeding tube in surgical patients requiring nasoenteral feeding.

## Methods

### Design

The CORE trial is an investigator-initiated, parallel-group, pragmatic, multicenter, randomized controlled non-inferiority trial. Patients will be randomly allocated to undergo EM guided or endoscopic nasoenteral feeding tube placement.

### Study population

All adult patients admitted to the gastrointestinal surgical wards in one of the five participating centers with an indication for post-pyloric enteral nutrition will be assessed for eligibility, regardless of the reason for admission (e.g. surgical procedures or conditions not requiring surgery).

### Inclusion criteria

Inclusion criteria are: admission to a gastrointestinal surgical ward; need for post-pyloric enteral nutrition via nasoenteral feeding tube as indicated by the treating physician and/or consulting dietitian (e.g. because of severe gastroparesis/gastric stasis not responding to prokinetics, intolerance of oral feeding due to gastroduodenal inflammation, postprandial pain or passage disorder due to swelling or outside pressure onto the duodenum or proximal enteric fistulae); and written informed consent.

### Exclusion criteria

Exclusion criteria are: age < 18 years; presence of a contraindication for enteral feeding; presence of a contraindication for EM guided placement (i.e. a history of esophageal varices, upper GI stenosis or obstruction, recent (<30 days) esophagectomy, or the presence of an implanted medical device that may be affected by the electromagnetic field of the EM guided system or vice versa (except for pacemakers and defibrillators) and necessity for tube placement during weekends or holidays.

### Randomization

**Figure 1:**
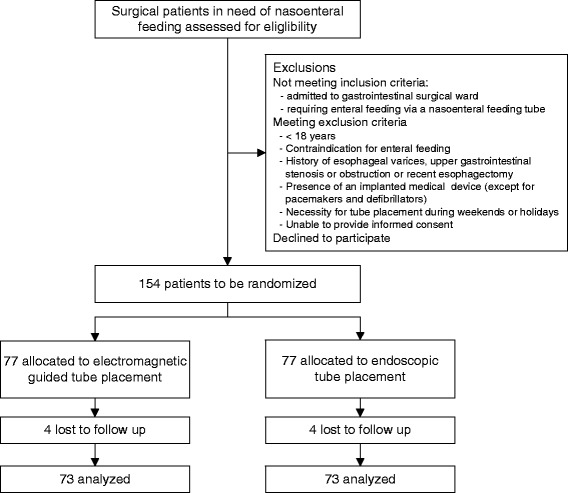
**Flow chart of participants in the CORE trial according to CONSORT.**

Eligible patients will be recruited on the clinical ward by the principal investigators or a designated substitute. After obtaining informed consent, patients will be randomized centrally by the study coordinator using an online randomization module (Clinical Research Unit, Academic Medical Center, Amsterdam, the Netherlands) in a 1:1 ratio between EM guided and endoscopic tube placement (Figure 1).

Randomization will be stratified by center to balance differences in general treatment regimens between hospitals and according to the presence of an altered upper GI (esophageal, gastric or duodenal) anatomy after previous surgery. Randomization is balanced for the presence of an altered upper GI anatomy because an altered anatomy might hamper feeding tube placement and decrease success rates. Permuted-block allocation is used to provide treatment allocation in equal proportions. The block size will be subject to random variation and concealed to all investigators involved in the study.

Blinding of patients and caregivers was considered practically unfeasible given the obvious differences between the two methods of tube placement (e.g. bedside placement or placement in the endoscopy department). Moreover, the primary outcome (reinsertion of an endoscope of tube) is sufficiently objective to minimize any potential risk of measurement bias. Feeding tubes are reinserted when oral intake is less than 50% of the patient’s daily required caloric intake. This decision will be made by the treating physician, whenever possible after consulting a dietitian.

### Group A: Electromagnetic guided nasoenteral feeding tube placement

**Figure 2:**
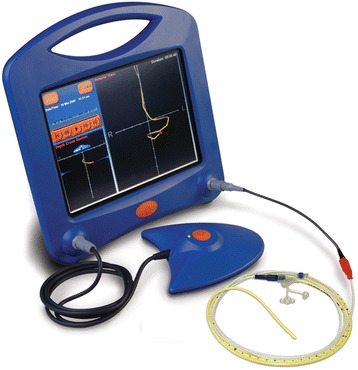
**The Cortrak® Enteral Access System (electromagnetic transmitting stylet, receiver unit and enteral feeding tube).** The tip of the tube is displayed on the monitor as a red dot and a yellow line reflects the path of the tube. *Image reproduced with permission of CORPAK MedSystems.*

The feeding tube (Corflo® nasojejunal feeding tube, Corpak Medsystems, Wheeling, Ill, US) is placed by a trained nurse at the patient’s bedside on the clinical ward, with the patient in a supine position. If a nasogastric decompression tube is in place, it is advised, but not mandatory, to empty the stomach and remove the nasogastric tube before proceeding. Preprocedural fasting is not required. By using an electromagnetic transmitting stylet, the nurse can follow the path of the tip of the feeding tube on a monitor screen (Cortrak® Enteral Access System, Corpak Medsystems, Wheeling, Ill, US) (Figure 2). The stylet at the tip of the feeding tube transmits its signal to a receiver unit placed at the patient’s epigastric region. Both the stylet and the receiver unit are attached by a cable to the monitor unit, which provides a graphic display of the feeding tube tip location and the followed track. In the anterior view, the tube can be followed on its way through the esophagus, stomach, duodenum, and jejunum. In the depth cross-section, the passage of the tube from the pylorus into the duodenal bulb and the second part of the duodenum can be seen. The tube is advanced to a post-pyloric position, preferably near or beyond the duodenojejunal flexure. Adequate positioning is assessed by the path of the tube on the screen. Subsequently, the transmitting stylet is removed from the feeding tube. Finally, the tube is secured to the nostrils with tape. Abdominal radiographs are not used, as the Cortrak® Enteral Access System was shown to correlate with abdominal radiography in 99.5% of cases and is cleared by the Food and Drug Administration (FDA) for feeding tube placement confirmation [9,11]. The stylet is kept at the patient’s bedside in case repositioning of the nasoenteral tube is required. The procedure is abandoned if the tip of the tube has not passed the pylorus after 30 minutes. In case of failure of EM guided placement, patients allocated to EM guided bedside placement will undergo endoscopic placement. According to the intention-to-treat principles these patients will be analyzed in the EM guided group.

### Group B: Endoscopic nasoenteral feeding tube placement

Endoscopy is performed by trained gastroenterologists (or supervised gastroenterologists in training) assisted by one or two endoscopy nurses in the endoscopy department. Patients fast from midnight but clear fluids are allowed up to 3 hours before the procedure. Conscious sedation is used if indicated (e.g. requested by the patient). A nasal or oral endoscope is introduced into the duodenum or jejunum. According to the gastroenterologist’s preference, the feeding tube is either pulled along with the endoscope, advanced through the endoscope or advanced over a guide wire and placed as far as possible in the duodenum or jejunum. Finally, the tube is secured to the nostrils with tape. Within 3 hours after tube placement, an abdominal radiograph is performed and reviewed by an independent radiologist who is not involved in the study. In case of incorrect feeding tube placement, repeat endoscopic tube placement is performed. In case of second placement failure, the study is terminated for that patient and an alternative feeding strategy (but not EM guided placement) is used according to the preference of the treating physician.

### General treatment regimen

After confirmation of the correct position of the feeding tube, enteral nutrition is initiated and increased to the required amount as advised by the treating physicians, whenever possible after consulting a dietitian. The dietitian can advise on a schedule for the resumption of oral feeding during enteral nutrition. When enteral nutrition is no longer indicated (i.e. oral intake exceeding 50% of the patient’s daily required caloric intake with an upward trend), it is ceased and the feeding tube is removed. These decisions are not influenced by the method by which the enteral feeding tube was placed.

In patients with symptoms of dislodgement (e.g. nausea, vomiting or reflux of tube feeding via the nasogastric tube), dislodgement of the nasoenteral feeding tube will be confirmed on abdominal radiograph or Cortrak® monitor (by reinsertion of the stylet). In patients with blockage of the tube (i.e. inability to pass tube feeding through the tube), an attempt will be made to resolve the clogging by flushing of the tube with water or acetylcysteine. In case of confirmed dislodgement or irreversible blockage of the tube, replacement (or repositioning if possible) will be performed with the allocated technique, except after conversion from EM guidance to endoscopy after failed initial EM guided placement. Prokinetic agents (e.g. metoclopramide or erythromycin) can be used according to the preference of the treating physician.

Patients are discharged when considered appropriate by the treating team. In select cases, patients can be discharged with the feeding tube in situ to continue enteral nutrition at home or a nursing home. In such cases, the care for enteral nutrition is taken over by (nursing) homecare. Any problems with the tube are taken care of in the outpatient clinic for as far as possible.

### Primary outcome

The primary outcome is the need for reinsertion of the feeding tube, defined as the insertion of an endoscope or tube in the nose/mouth and esophagus for (re)placement of the feeding tube. This outcome reflects the feasibility of the technique itself as well as the need for repeated procedures due to tube-related complications. Tubes will be reinserted when there is an ongoing indication for enteral feeding (as advised by the consulting dietitian) after initial unsuccessful placement, dislodgement or blockage of the tube. Endoscopic placement after failed initial EM guided attempt (crossover) is also considered a reinsertion. Repositioning guided by the EM transmitting stylet is not considered a reinsertion as long as the tube has not left the stomach/esophagus.

### Secondary outcomes

EM guided placement of a nasoenteral feeding tube is hypothesized to be less discomforting to the patient compared to endoscopic placement, at lower costs. Secondary outcomes therefore include: patient-reported outcomes (e.g. pain, discomfort, and total burden) and costs of both tube placement procedures. The costs will include the direct and indirect medical costs of initial tube placement (including personnel, materials, sedation, equipment and transport), tube related complications, diagnostic investigations, and feeding related interventions.

Other secondary outcomes are: the success rate of tube placement; duration of the tube placement procedure; time between physician order and tube placement and start of feeding; time to reach the feeding goal; all feeding related interventions (including EM guided repositioning without reinsertion of the tube) with corresponding indication; duration of tube stay; tube (placement) related complications (such as dislodgement, blockage, epistaxis, aspiration); use of parenteral nutrition; length of hospital stay; and in-hospital mortality (see Table 1 for definitions).

**Table 1:** **Definitions of outcomes**

**Outcome**	**Definition**
Reinsertion	The insertion of an endoscope or tube in the nose/mouth and esophagus for (re) placement of the feeding tube.
Repositioning	Reinsertion of the EM transmitting stylet when the tube has not left the stomach/esophagus.
Dislodgement	Any displacement of the feeding tube making continuation of tube feeding unsafe (e.g. because it is delivered into the stomach in the presence of gastroparesis) or impossible (e.g. when the tube has been removed from the patient), confirmed on the Cortrak® monitor or abdominal radiograph.
Blockage	The inability to pass tube feeding through the tube whilst it is still in the correct position.
Successful tube placement	The tip of the feeding tube positioned beyond D2 [preferably near the ligament of Treitz (i.e. duodenojejunal flexure)] or in the efferent jejunal limb (in the presence of a gastro- or duodenoenterostomy) on the Cortrak® monitor or abdominal radiograph (depending on the placement method) followed by successful enteral feeding, without signs of feeding entering the stomach.
Duration of tube stay	The number of days that the feeding tube was in its correct position.

### Data collection and follow-up

**Figure 3:**
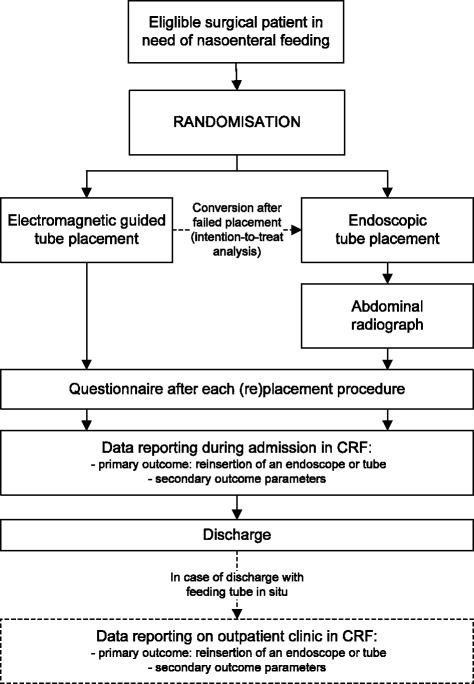
**CORE trial flow chart of eligibility and group allocation.**

Clinical data with regard to baseline characteristics and outcomes will be collected during hospital admission using written standardized case report forms (CRFs) (Figure 3). The CRFs will be completed by the local treating physicians or the study coordinator. The CRFs will be checked with source data by the primary and/or secondary study coordinator. The utilization of healthcare in terms of direct medical costs of initial tube placement, sedation, tube-related complications, diagnostic investigations, and feeding related interventions will be registered as part of the data collection for the trial.

Patients will be asked to complete a short questionnaire after each tube (re)placement procedure. The questionnaire will consist of a visual analogue scale (VAS) scoring sheet, for the dimensions discomfort, pain, social embarrassment, anxiety, and total burden (comparable to the study by Deutekom *et al*. [16]). In addition, patients will also be asked what their advice would be to a friend or colleague in the same situation. Patients randomized to EM guided tube placement with past experiences with endoscopic tube placement and vice versa will additionally be asked for their preference for one or the other tube placement method.

Patients are followed for as long as they are hospitalized and during any outpatient clinic or day-care visit related to the feeding tube (see Additional file 1 for the time schedule of enrolment, interventions, and assessments).

### Safety

All physicians involved in the study will be instructed to contact the coordinating investigator in case of mortality or tube (placement) related unexpected adverse events leading to prolonged hospitalization. All severe adverse events that may be related to the feeding tube or placement procedure and all cases of mortality will be reported to the Central Committee on Research involving Human Subjects (in Dutch: Centrale Commissie Mensgebonden Onderzoek) and the Institutional Review Board using an online module [17]. Due to the perceived negligible risk associated with participation in this study, monitoring by a Data Safety Monitoring Board is not required according to Dutch legislation.

### Ethics

The study will be conducted according to the principles of the Declaration of Helsinki (59th version, October 2008) and in accordance with the Dutch Medical Research Involving Human Subjects Act (WMO). The independent ethics review board of the Academic Medical Center (Amsterdam, the Netherlands) has approved the study protocol. Secondary approval was obtained from the boards of the Canisius Wilhelmina Hospital, Gelre Hospital, Hospital Gelderse Vallei, and University Medical Center Utrecht according to the Dutch CCMO External Review Directive 2012 (RET 2012). Written informed consent will be obtained from all participating patients prior to randomization. The trial is registered in the Dutch Trial Register (http://www.trialregister.nl/trialreg/index.asp) with identification number NTR4420.

### Statistical aspects

#### Sample size calculation

The CORE trial is designed as a non-inferiority trial, hypothesizing that the need for reinsertion of a nasoenteral feeding tube after EM guided placement is at most 10% worse as compared to endoscopic placement.

Sample size is calculated based on data from previous studies and experiences from our pilot study.

Success is defined the absence of the need for reinsertion of an endoscope or tube. We estimate a success rate of 78% in the EM guided group versus 70% in the endoscopic group. When the non-inferiority limit is set at 10%, significance level at 0.05 and power at 80%, the sample size required per study arm is 73 [18]. Taking into account a 5% loss to follow-up rate (based on previous studies), a total of 154 patients will have to be randomized in this study.

#### Descriptive statistics

For dichotomous data, frequencies will be presented. Continuous data will be presented as means and standard deviations or medians and interquartile ranges, depending on their distribution. Baseline characteristics (all prior to randomization) include: age, sex, body mass index, American Society of Anesthesiologists (ASA) physical status, indication for hospital admission, type of surgery (if applicable), indication for enteral nutrition, cause of gastroparesis (if applicable), interval between surgery and primary tube placement, use of prokinetic agents and presence of an altered upper GI anatomy after previous surgery.

#### Analyses

All analyses will be according to the intention-to-treat principles, meaning that all randomized patients are included in their initially assigned study arm, regardless of adherence to study protocol. The primary and secondary outcomes will be compared between the treatment groups. Results are presented as risk ratios with corresponding 95% confidence intervals. Except for the primary analysis of non-inferiority, a difference with a two-tailed P < 0.05 is considered statistically significant. In the event of imbalance between the two study arms a multivariable logistic regression will be used to correct for possible confounders. Additional as-treated and per-protocol analyses will be performed for the primary endpoint and the success rate of both tube placement methods.

A predefined subgroup analysis will be performed in patients with and without an altered upper GI anatomy after previous surgery. We will use logistic regression models to perform a formal test for interaction to assess whether outcomes differed significantly between these subgroups.

Interim analysis to evaluate serious adverse events and potentially prematurely terminate the study will not be performed, because of the perceived negligible risk associated with participation in this study.

### Dissemination policy

The trial’s results will be submitted to a peer-reviewed journal regardless of the outcome. Co-authorship will be based on the international guidelines with a maximum of three co-authorships per participating center. Participating clinicians that do not fulfill these criteria will be listed as ‘collaborator’ and the journal will be asked to present the names of all collaborators to be listed in PubMed. The order of authors will be based primarily on scientific input and secondarily on the number of randomized patients.

## Discussion

The CORE trial is initiated by gastrointestinal surgeons and gastroenterologists based on the potential benefits of EM guided tube placement (e.g. reduced patient discomfort and costs). The study is designed as a multicenter, randomized controlled non-inferiority trial to answer the question whether EM guided nasoenteral feeding tube placement is at least as effective as endoscopic placement in surgical patients. Superiority would, of course, be preferred, but such a trial would be practically impossible given the required sample size of nearly a thousand patients. Moreover, superiority regarding effectiveness is not required, since EM guided nasoenteral tube placement may still be recommended if its effectiveness is similar, or at least not worse than the non-inferiority limit, to endoscopic tube placement, because of the intrinsic advantages of this technique [19].

In contrast to previous studies on EM guided nasoenteral feeding tube placement, the CORE trial is an investigator-initiated randomized controlled multicenter trial comparing EM guided placement to the current conventional tube placement method (endoscopy). Moreover, it does not only focus on successful tube placement, but also on patient-reported outcomes and costs. When looking at effectiveness, the success rate of initial tube placement would have been the most obvious primary outcome. However, since passage of the tube through the naso- and oropharynx is considered to be the most burdensome part of tube placement, a reduction in reinsertions of the tube seems much more relevant to the patient. In addition, the success of tube placement is included in this outcome because reinsertion is required after failure. Furthermore, EM guided tube placement might lead to a slight reduction in necessary reinsertions, since tubes dislodged into the stomach can be repositioned without removal of the tube (as is the case with endoscopic replacement).

The study population consists of patients admitted to gastrointestinal surgical wards who are in need of nasoenteral feeding whereas previous randomized controlled trials (and the majority of cohort studies) have only been performed in critically ill patients. This population is chosen because the incidence of gastroparesis is relatively high in these patients, since their GI system is frequently disordered by an operation, postoperative complications or the underlying or concomitant disease. Gastroparesis is especially a problem in patients after abdominal surgery and many feeding strategies have been investigated [20-22]. The high incidence of gastroparesis may not only lead to a frequent need for nasoenteral feeding, but it also hampers post-pyloric tube placement due to gastric stasis. Moreover, in a substantial subset of surgical patients the upper GI anatomy is altered due to surgery, which may complicate tube placement as, consequently, also the route of the feeding tube has changed. When a nasoenteral feeding tube placement method would prove to be successful in the surgical population, the results can probably be extrapolated easily to the overall hospital population requiring nasoenteral feeding.

In conclusion, the CORE trial is a randomized controlled multicenter trial, which will generate evidence on the effectiveness of EM guided placement of nasoenteral feeding tubes in surgical patients and its related costs as compared to endoscopic placement, and can potentially offer a strong argument to further implement this technique as method of choice for placement of nasoenteral feeding tubes.

## Trial status

The first patient was randomized on the 13th of March 2014. As of February 23rd 2015, 133 of 154 patients (86%) have been randomized and inclusion is progressing according to schedule.

## Additional file

Additional file 1:
**Time schedule of enrolment, interventions, and assessments of participants in the CORE trial.**

## Abbreviations

ASA: American society of anesthesiologists
CORE: Electromagnetic guided or endoscopic nasoenteral feeding tube placement in surgical patients
CRF: Case report form
EM: Electromagnetic
GI: Gastrointestinal
VAS: Visual analogue scale
