# Supplementary material for: Electromagnetic guided bedside or endoscopic placement of nasoenteral feeding tubes in surgical patients (CORE trial): study protocol for a randomized controlled trial
Source: Trials. 2015 Mar 26;16:119. doi: 10.1186/s13063-015-0633-1 (PMC4390000; doi:10.1186/s13063-015-0633-1)
Supplement: Additional file 1: — Time schedule of enrolment, interventions, and assessments of participants in the CORE trial. [file 13063_2015_633_MOESM1_ESM.doc]

Additional file 1

Time schedule of enrolment, interventions, and assessments of participants in the CORE trial.

|  | Enrolment and Allocation | Post-allocation | | Close-out |  |
| --- | --- | --- | --- | --- | --- |
| Time point | Baseline | Initial placement | Replacement (or repositioning) | Discharge | Replacement after discharge |
| ENROLMENT: |  |  |  |  |  |
| Eligibility screening | X |  |  |  |  |
| Informed consent | X |  |  |  |  |
| Allocation | X |  |  |  |  |
| INTERVENTIONS: |  |  |  |  |  |
| Group A: Electromagnetic guided placement |  | X | X |  | X |
| Group B: Endoscopic placement followed by abdominal radiography |  | X | X |  | X |
| ASSESSMENTS: |  |  |  |  |  |
| Age; | X |  |  |  |  |
| Sex; |
| Body Mass Index; |
| ASA physical status; |
| Indication for hospital admission; |
| Type of surgery; |
| Indication for enteral nutrition; |
| Cause of gastroparesis; |
| Interval between surgery |
| and primary tube placement; |
| Use of prokinetic agents; |
| Presence of an altered upper GI anatomy |
| Success of procedure; |  | X | X |  |  |
| Duration of procedure; |
| Tube placement related complications |
| Time between physician order and tube placement; |  |  |  | X |  |
| Time between physician order and start of feeding; |
| Time to reach the feeding goal; |
| Duration of tube stay; |
| Tube related complications; |
| Use of parenteral nutrition; |
| Length of hospital stay; |
| In-hospital mortality |
| Questionnaire (discomfort, pain, social embarrassment, anxiety, and total burden) |  | X | X |  | X |
